# Case report: rapidly fatal bowel ischaemia on clozapine treatment

**DOI:** 10.1186/1471-244X-6-43

**Published:** 2006-10-19

**Authors:** Giles Townsend, David Curtis

**Affiliations:** 1 East London and City Mental Health Trust, Department of Adult Psychiatry, Royal London Hospital, Whitechapel, London E1 1BB, UK

## Abstract

**Background:**

There have been previous reported deaths due to clozapine-induced constipation. In all these cases patients have experienced prior abdominal symptoms over a period of weeks or months.

**Case presentation:**

We report the sudden death due to constipation of a healthy young male patient on clozapine without any known history of prior abdominal symptoms.

**Conclusion:**

Psychiatrists need to be alert to the medical emergencies which can occur in the context of clozapine treatment and also need to make other clinicians who may have contact with their patients aware of these.

## Background

There have been six previously published cases of death secondary to clozapine-induced constipation [1-3]. Of these, two patients died from faecal peritonitis, two from aspiration of faeculent vomitus as a result of bowel obstruction and two from bowel necrosis. In all these cases there had been prior complaints of constipation and/or other abdominal symptoms for weeks to months before the fatal event. Here we describe a case of constipation, presumably clozapine-induced, where death from bowel ischaemia occured within 2 days from the first complaint of constipation and without any prior reported abdominal symptoms which might have provided a warning to the clinicians involved.

## Case presentation

A 20-year-old male with a year long history of schizophrenia which had been unresponsive to trials of two atypical antipsychotic drugs was commenced on clozapine. The dose was titrated over the next year to 900 mg daily. Due to persisting negative symptoms amisulpiride 400 mg twice daily was added with good response after one month. The patient was reviewed regularly over the next year, continued to improve and did not report any side effects to members of the multidisciplinary mental health team working to support him in the community. He appeared to be fit and healthy. Although he usually lived in supported accommodation he was staying temporarily with his family and from their account he complained of having constipation for 2 days before presenting to his GP with severe abdominal pain. He was prescribed medication and returned home but his condition deteriorated further and a few hours later an ambulance was called. He collapsed and died before reaching hospital. Post mortem examination revealed that he had impacted faeces which had pressed against the bowel wall causing ischaemia. This had led to infarction of this part of the bowel.

## Conclusion

This case demonstrates that death can occur over a very short time course from constipation, in this case presumably induced by clozapine. Death from constipation and subsequent bowel infarction is relatively common in elderly patients and infarction causes a far more rapid and dangerous deterioration than does intestinal obstruction. In the present case this meant that this patient did not have any contact with psychiatric services between the onset of his symptoms and his rapid demise, in spite of regular follow-up. Although the risk of neutropenia is relatively well-known, it should be borne in mind that clozapine is reported to be associated with a number of other syndromes which may be rapidly fatal including not only constipation and obstruction but also cardiovascular collapse, seizures and ketoacidosis. Psychiatrists working with such patients should not only themselves be vigilant regarding such complications but should take steps to see that other clinicians to whom the patient may present are also aware of them.

## Competing interests

The author(s) declare that they have no competing interests.

## Authors' contributions

Both authors were equally involved in the preparation of this manuscript.

## Pre-publication history

The pre-publication history for this paper can be accessed here:


